# GC-MS Analysis and Gastroprotective Evaluations of Crude Extracts, Isolated Saponins, and Essential Oil from *Polygonum hydropiper* L.

**DOI:** 10.3389/fchem.2017.00058

**Published:** 2017-08-02

**Authors:** Muhammad Ayaz, Muhammad Junaid, Farhat Ullah, Abdul Sadiq, Muhammad Shahid, Waqar Ahmad, Ihsan Ullah, Ashfaq Ahmad, Nawazish-i-Husain Syed

**Affiliations:** ^1^Department of Pharmacy, University of Malakand Chakdara dir, Pakistan; ^2^Department of Pharmacy, University of Peshawar Peshawar, Pakistan; ^3^Department of Pharmacy, Sarhad University of Information Technology Peshawar, Pakistan; ^4^Department of Pharmacy, University of Swabi Swabi, Pakistan; ^5^Department of Pharmacology, University College of Pharmacy, University of Punjab Lahore, Pakistan

**Keywords:** *Polygonum hydropiper*, *Canavalia ensiformis*, indophenol method, urease, ulcerogenesis, *Proteus mirabilis*

## Abstract

Peptic ulceration is among the most prevalent gastrointestinal disorders characterized by pepsin and gastric acid mediated mucosal damage, as result of imbalance between defensive and offensive processes. The main objective of the current study was to investigate the antiulcer potentials of *Polygonum hydropiper* crude methanolic ectract (Ph.Cr) in aspirin induced ulcerogenesis using pylorus ligated rat model. *In-vitro* urease and *Proteus mirabilis* inhibitory potentials were evaluated using standard protocols. All fractions were analyzed using GC-MS to identify major components. The aspirin induced ulcerogenesis in pylorus ligated rat model was associated with significant changes in the mean ulcer score [*F*_(5, 30)_ = 7.141, *P* = 0.0002], gastric juice volume [*F*_(5, 30)_ = 8.245, *P* < 0.0001], gastric juice pH [*F*_(5, 30)_ = 5.715, *P* = 0.0008], free acidity [*F*_(5, 30)_ = 4.544, *P* = 0.0033], total acidity [*F*_(5, 30)_ = 2.740, *P* = 0.0373], and pepsin concentration [*F*_(5, 30)_ = 2.335, *P* = 0.0664]. Pre-treatment with Ph.Cr at 100, 200, and 400 mg/kg dose exhibited marked gastroprotective and anti-ulcerogenic effect in the aspirin induced pyloric ligation ulcerogenesis model at 100, 200, and 400 mg/kg as indicated by ulcerative biochemical parameters. In urease inhibition assay, leaves essential oil (Ph.Lo), saponins (Ph.Sp), and chloroform extract (Ph.Chf) exhibited highest activities with IC_50_ of 90, 98, and 520 μg/ml, respectively. Ph.Sp, Ph.Chf, ethyl acetate (Ph.EtAc), and Ph.Cr showed MICs of 25, 30, 32.25, and 40.50 μg/ml, respectively against *P. mirabilis*. Several compounds were identified in GC-MS analysis of samples. Significant *in-vivo* antiulcer, urease inhibitory as well as anti-proteus potentials of *P. hydropiper* solvent extracts, signify its potential use for the management of peptic ulcers and may provide scientific bases for the traditional uses of the plant.

## Introduction

Peptic ulcer disease is a major gastrointestinal disorder which results from an imbalance between defensive (mucus secretion, alkaline and neutral pancreatic and biliary juices, intact mucosal barrier, gastroprotective prostaglandins) and offensive mechanisms (hydrochloric acid, pepsin, refluxed bile, leukotrienes, reactive oxygen species, *Helicobactor pylori* infection, ischemia). The disease has high prevalence and is associated with significantly high annual mortality rates (Yuan et al., 2006; Malfertheiner et al., 2009). There are number of drugs clinically available to counteract peptic ulcer disease, however these drugs have their characteristic side effects and complications that hinder their frequent use, therefore limiting their clinical effectiveness (Henry and Langman, 1981; Abraham, 2012). Natural products, especially plants derived chemicals are considered as promising source for the development of new agents with safe therapeutic window (Atanasov et al., 2015). Traditional medicine using plants have been shown to be successful in the treatment of gastrointestinal disorders including peptic ulcer disease (Gadekar et al., 2010). Consequently, plant extracts stand out as the most promising substances in the search for new therapies for the treatment of gastric ulcer. In this regard, numerous pharmacological agents with known anti-ulcer activity have been isolated from potential plant extracts (Lewis and Hanson, 1991). Clinical research has confirmed the efficacy of several plants for the treatment of gastroduodenal diseases (Kanner and Lapidot, 2001; Joo, 2014). Thus, there is an ample scope of plant extracts available for screening of potential remedy for peptic ulcer disease.

Currently, several models are available for the anti-ulcer evaluations of the test compounds from natural and synthetic sources (Adinortey et al., 2013). Yet, the selection of an appropriate model is very difficult owing to the associated advantages and disadvantages of these models. Among the *in-vivo* anti-ulcer procedures, pylorus ligation induced ulcer model is extensively used. Stomach pylorus ligation leads to gastric acid accumulation causing autodigestion of mucosal layer and induction of ulcers. This model predict cytoprotective effects of test compounds by enhancing the protective mucous secretion and inhibition of acid and pepsin (Vogel and Vogel, 1997). Animals are maintained on fasting for 36–72 h before pylorus ligation and subsequently ligated using “Shay” procedure under anesthesia (Shay, 1945). Test samples are orally administered 1 h before ligation and animals are evaluated for ulcer induction after 18–20 h post drugs administration.

Ureases are important group of enzymes implicated in the catabolism of urea leading to the production of ammonia and carbamic acid (Mobley and Hausinger, 1989). Urease enzyme serves as a virulence factor and is responsible for pathogenesis in humans like ammonia encephalopathy, pyelonephritis, urolithiasis, urinary catheter encrustation, and hepatic coma. Additionally, high concentration of ammonia disturb mucosal permeability, particularly hydrogen ions passage across the mucosal surface and causes formation of peptic ulcers (Khan et al., 2010). *H. pylori* secrete a potent urease which catalyzes the hydrolysis of urea to produce ammonia which alkalinize the stomach environment and thus enable this microorganism to survive. Urease from *H. pylori* has been recognized as a potential therapeutic target for the management of peptic ulcer. Consequently, inhibitors of this enzyme have got much attention as potential anti-ulcer agents.

*Proteus mirabilis* is the most common pathogen concerned with stone formation (Rosenstein and Griffith, 1986). With alkalinization of urine, the soluble polyvalent ions become supersaturated and this happens when ammonia is released by microbial urease-catalyzed urea hydrolysis leading to crystallization and stone formation. *P. mirabilis* is the primary urease-producing pathogen in humans and its role in pyelonephritis has been well-established both in rat model as well as in tissue culture system (Braude and Siemienski, 1960; Musher et al., 1975). *Proteus* urease also play important role in the etio-pathogenesis of rheumatoid arthritis (Rashid and Ebringer, 2007).

*Polygonum hydropiper* is traditionally used to treat dyspepsia, diarrhea, menorrhagia, bleeding disorders and to strengthen fragile blood vessels (Ayaz et al., 2014a). Recently, we reported AChE/BChE inhibitory, antioxidant, and cytotoxic potentials of *P. hydropiper* (Ayaz et al., 2014a, 2016b). Based on the ethnomedicinal uses of this plant and related species, this study was designed to investigate phytochemical analysis and gastroprotective potentials of *P. hydropiper*.

## Materials and methods

### Chemicals and drugs

Urease from *Canavalia ensiformis* (Jack bean) (SLBB0100V Sigma Aldrich), thiourea (CAS 62-56-6, Sigma Aldrich), sodium nitroprusside (CAS 13755-38-9, Sigma Aldrich CHEMIE GmbH USA), ceftriaxone (Geltis, Shaigan Pharmaceuticals, Pakistan), sodium carbonate (Merck, USA), 2,4,6-tri(2-pyridyl)-1,3,5-triazine Alfa Aesar, CAS (3682-35-7, Germany) and solvents of analytical grade were purchased from distributor of Sigma Aldrich in Pakistan.

### Plant material processing

Whole plant of *P. hydropiper* was acquired from Talash Valley, Dir (L), Khyber Pakhtunkhwa (KP), Pakistan in the month of July, 2013 as we reported previously (Ayaz et al., 2014a, 2016b). The identification of plant was done by Dr. Gul Rahim, and subsequently a voucher specimen was maintained with Voucher No. H.UOM.BG.107 at the University of Malakand, herbarium for future record purpose. The plant material was dried in shade with subsequent pulverization and fractionation. The whole process was carried following standard protocol as we reported previously (Ayaz et al., 2014b; Ullah et al., 2016; Ali et al., 2017).

### Crude saponins extraction

Crude saponins were isolated from dried plant powder as we reported for this plant and other natural products (Ayaz et al., 2014a, 2016b; Zeb et al., 2014; Ahmad et al., 2015). Briefly, 60 g of powder plant material was transferred to conical flask and 100 ml of 20% ethanol was added to it. The resultant mixture was slowly heated for 4 h at 55°C using water bath. The mixture was filtered and again extracted with 200 ml of ethanol. The volume from both extractions were combined and concentrated with the help of water bath. Finally, 40 ml was transferred to separating funnel and 20 ml of diethyl ether was added to it with vigorous shaking. The Diethyl ether layer was discarded and to the aqueous fraction, 60 ml of *n*-butanol was added. The resultant aqueous-butanol mixture was passed through 5% NaCl solution and solvents were evaporated to obtain crude saponins (Zeb et al., 2016).

### Essential oil isolation

We previously reported isolation of essential oils from *P. hydropiper* (Ayaz et al., 2015a). Briefly, fresh collected leaves of *P. hydropiper* were washed with distilled water and subsequently hydrodistilled with the help of a Clevenger apparatus (Clevenger, 1928). The resultant oils were condensed, collected, and any water content was removed via anhydrous sodium sulfate (Bassole et al., 2011; Ayaz et al., 2017). The oils were transferred to glass vials, tightly sealed and refrigerated at −30°C prior to further analysis.

### Gas chromatography–mass spectrometry (GC/MS) analysis

Samples were analyzed via gas chromatograph (Agilent USB-393752) equipped with FID detector and capillary column as per specifications we published previously (Ayaz et al., 2015a; Ahmad et al., 2016).

### Phytoconstituents identification

Components of the samples were identified by comparing their retention times with previously known compounds in the literature and from the spectral data acquired from NIST and Wiley and NIST libraries. Moreover, the fragmentation pattern of the mass spectra was also compared with already published data for further authentication (Stein et al., 2002; Adams, 2007).

### Urease inhibition assay

Urease inhibitory potentials of the test samples were evaluated following previously reported procedure (Weatherburn, 1967). In brief, 25 μl enzyme solution and 55 μl of buffer (containing 100 mM urea) were incubated with 5 μl of test solution (1 mM concentration) in micro plate reader (room temperature for 15 min). Enzyme inhibitory potentials of our test samples were assessed from the rate of ammonia production. After 50 min of incubation, absorbance was recorded at 630 nm using Uquant Micro plate reader (BioTek Instruments Highland Park Winooski SN 213541 USA) using thiourea as control drug. Percent enzyme inhibition was calculated as;


$$\%\;\text{ inhibition}=\frac{100-(\text{OD}\;\text{ test}\;\text{ well})}{\text{OD}\;\text{ control}}\times 100$$

### Anti-*proteus mirabilis* activity

#### Bacterial culture standardization

*Proteus mirabilis* was grown overnight at 37°C in BOD incubator HYSC Korea (BI-81/150/250). Bacterial cultures were diluted in distilled water and were adjusted to cell density of 1 × 10^8^ CFU/ml by comparing with McFarland standard. The preparation was further diluted corresponding to 1 × 10^6^ CFU/ml cell density via UV visible spectrophotometer (Thermo electron corporation USA) at 625 nm (Ayaz et al., 2015c).

#### Disc diffusion procedure

Anti-Proteus activity of our test samples were evaluated following previously reported disc diffusion procedure (Ayaz et al., 2015b,c). Sterilized nutrient agar plates were inoculated with *P. mirabilis* preparation under laminar and sterile filter discs loaded with test samples were placed on the surface of plates. DMSO loaded and standard ceftriaxone discs were used as negative and positive controls respectively. All plates were incubated overnight at 37°C and diameters of inhibitory zone (DIZ) were measured around the discs. The diameters of zone of inhibition produced by the extracts were then compared with the standard antibiotic ceftriaxone.

#### Well diffusion assay

Anti-Proteus potentials of our samples were also investigated using previously reported well diffusion assay (Ayaz et al., 2016a). Pre-sterilized agar plates were inoculated with the bacterial preparation and 6 mm wells were made equidistantly on the surface using sterile cork borer. Subsequently, 100 μl of test solution were added to the wells and incubated overnight at 37°C. DIZ were measured around the bore for test samples and expressed in mm as mean ± SEM (*n* = 3).

#### Determination of MICs

MICs were determined by nutrient broth as well as agar dilation techniques following clinical and laboratory standard institute (CLSI) guidelines (National Committee for Clinical Laboratory Standards, 1993). Test samples (2–512 μg/ml) were transferred to pre-sterilized broth in tubes and subsequently inoculated with *P. mirabilis*. All tubes were overnight incubated at 37°C using shaker incubator. In agar dilution technique sample solutions were added to nutrient agar and bacterial strains were spot-inoculated on the surface of nutrient agar with consequent incubation at the same conditions. The concentration of test samples where no visible microbial growth was observed was considered as MIC. Experiments were performed in triplicates.

### *In vivo* evaluation of gastroprotective activity

#### Animals

Adult male Sprague-Dawley rats (150–200 g) were purchased from the National Institute of Health (NIH), Islamabad, Pakistan. The animals were maintained under standard laboratory conditions in polypropylene cages under 12 h light/dark cycle, controlled temperature (24 ± 2°C), fed with commercial pellet diet, and water *ad libitum*. The experimental procedures on animals were in accordance with the NIH guidelines for the care and use of laboratory animals and conformed to the Animal Research: Reporting *In vivo* experiments (ARRIVE) guidelines.

#### Ethical approval

The data presented in this manuscript belong to the thesis of Dr. Muhammad Ayaz, conducted at Department of Pharmacy, University of Malakand, Pakistan. All experiments were performed according to the rulings of the Institute of Laboratory Animal Resources, Commission on Life Sciences, National Research Council (1996) (Clark, 1996). The study protocol was approved by departmental research ethics committee (DREC), Department of Pharmacy, University of Malakand via reference no DREC/20160502/01. All animals were provided food and water ad libitum.

#### Induction of ulcerogenesis and treatment protocol

The aspirin induced ulcerogenesis in pylorus ligated rat model was used for the evaluation of gastroprotective activity of Ph.Cr (Umamaheswari et al., 2007). The extract, aspirin, and the positive control ranitidine were prepared in 1% sodium carboxy methyl cellulose suspension as vehicle and were administered orally. The animals were divided into six groups with each group consisting of six animals (*n* = 6). Group I received only vehicle and served as negative control. Group II received only aspirin (200 mg/kg) and represented the ulcerated control. Group III is the drug treated positive control and received ranitidine (50 mg/kg). Group IV, V, and VI were treated with the Ph.Cr extract at doses of 100, 200, and 400 mg/kg, respectively. Each animal group was administered respective doses once a day for consecutive 4 days and on the last day, all the drug treated groups received aspirin orally at a dose of 200 mg/kg, 2 h after the administration of respective drugs treatment. Each animal was fasted for 18 h after the respective assigned treatment and was anesthetized with ketamine hydrochloride (100 mg/kg, i.p.), the abdomen was opened, and the pyloric end of the stomach was ligated with thread, taking care of the blood vessels. The abdomen was closed and animals were kept individually in a platform with a wide mesh wire gauge to prevent coprophagy. Gastric juice was allowed to collect for 4 h after pyloric ligation. After 4 h of pyloric ligation, rats were sacrificed and their abdomen was opened and the cardiac end of the stomach was ligated. The stomach was quickly dissected out and cut open along the greater curvature and gastric juice was collected and centrifuged (2,500 g, 5 min) to obtain clear gastric juice.

#### Assessment of gastroprotection

Ulcer scoring was graded as 0 = almost normal mucosa; 0.5 = red coloration; 1 = spot ulcers; 1.5 = hemorrhagic streaks; 2 = ulcers ≥ 3 mm but ≤ 5 mm; and 3 = ulcers > 5 mm. The volume of the collected gastric juice was measured in a graduated cylinder. Gastric juice pH was determined using a digital pH meter. Free and total acidity were estimated according to Srivastava et al. (2010) and were expressed as mEq/L. Pepsin activity was measured according to the method of Kalra et al. (2011) and was expressed as μg/mL. For histological assessment, the isolated stomachs were subjected to tissue processing and staining with hematoxylin and eosin (H & E) for histopathological examination which were recorded with 100x lenses.

### Assessment of IC_50_

Concentrations of test samples which exhibited 50% inhibition against the test enzymes (IC_50_) were calculated from dose-response curve for urease inhibition assay.

### Statistical analysis

All assays were repeated three times and results obtained presented as means ± SEM. One way ANOVA followed by multiple comparison Dunnett's test was applied to the data for the comparison of test samples with standard drugs using GraphPad software. *P* < 0.05 were considered as statistically significant.

### Cluster analysis

An IC_50_ based cluster analysis was performed and dendrogram was developed for test samples via SPPS software version 16.0 using Ward's procedure to make hierarchical clusters.

## Results and discussion

### GC-MS analysis

**Figure 1:**
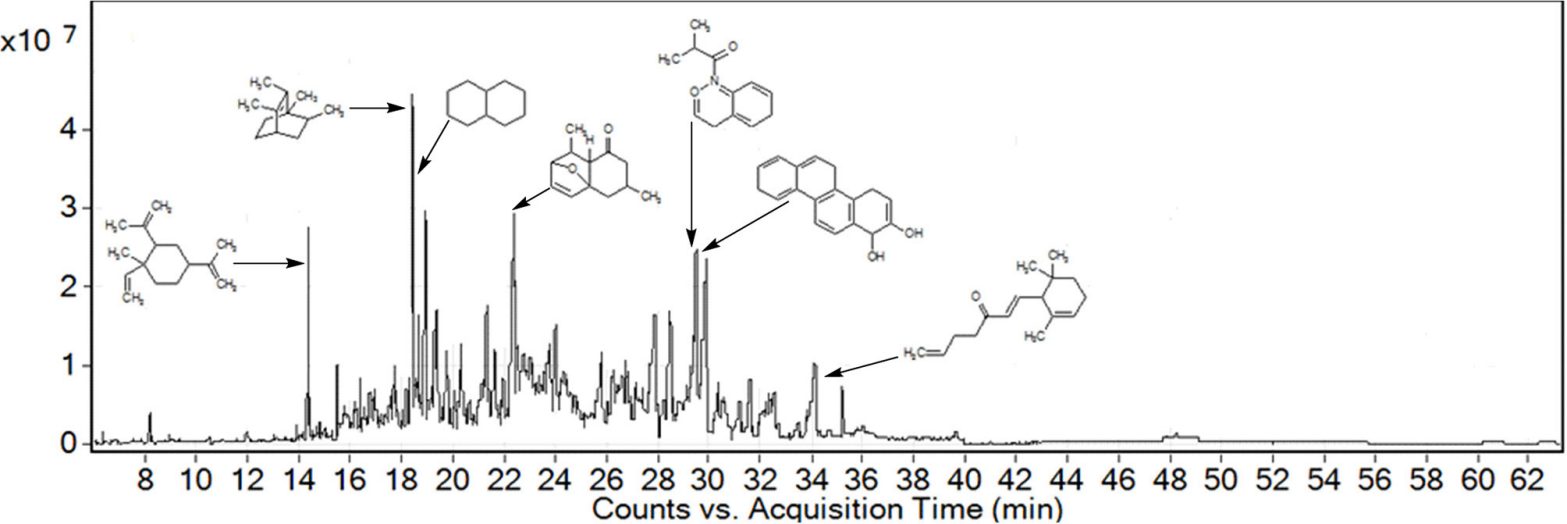
GC-MS chromatogram of Leaf essential oil from *Polygonum hydropiper* showing major identified compounds.

GC followed by GC-MS analyses were performed to identify major compounds in various fractions and essential oil isolated from *P. hydropiper*. In the phytochemical analysis of Ph.Cr, Ph.Hex, Ph.Chf, Ph.Bt, Ph.EtAc, and Ph.Lo the total number of identified compounds were 126, 124, 181, 131, 164, and 141, respectively. Among the identified compounds in Ph.Cr, 2,3-dihydro benzofuran, neophytadiene, 3,7,11,15-tetramethyl-2-hexadecen and 1-dodecanol, 3,7,11-trimethyl-hexa-hydro-farnesol were found in highest concentrations of 7.89, 25.2, 10.71, and 8.14%, respectively (Table 1). In analysis of Ph.EtAc, coumaran (34.05%), p-vinylguaiacol (16.94%), and alpha santolina alcohol (13.37%) were found in major concentrations. Similarly, in analysis of Ph.Hex, humulene oxide (13.79%), 9,12,15-octadecatrienoic acid, methyl ester (8.85%), methyl palmitate (7.68%), drimenol (7.26%), and caryophyllene oxide (7.7%) were the compounds found abundantly. Methyl linolenate, methyl palmitate, and 9,12-octadecadienoic acid methyl ester were present in higher concentrations of 10.47, 10.46, and 7.23%, respectively in Ph.Chf. Furthermore, coumaran (53.32%), p-vinylguaiacol (17.44%), borneol (14.21%), alpha-santolina alcohol (4.45%), and aristolone (4.01%) were major identified phyto-constituents in Ph.Bt. Among the identified compounds of Ph.Lo, decahydronaphthalene, and bicyclo [2.2.2]oct-2-ene, 1,2,3,6-tetramethyl were highly abundant compounds with 38.29 and 36.33% composition, respectively (Figure 1).

**Table 1:** Phytochemical analysis and Identification of major compounds in various fractions of *Polygonum hydropiper*.

**RT**	**Height**	**Height %**	**Area**	**Area %**	**Area sum %**	**Base peak m/z**	**Width**	**Compound name**
**(A)**								
10.341	818,728	33.34	3E + 06	31.32	7.89	120	0.198	2,3-dihydro benzofuran
18.807	582,512	23.72	1E + 06	14.06	3.54	43.1	0.09	Humulene oxide
19.222	570,159	23.22	1E + 06	14.61	3.68	67.1	0.087	(−)Caryophyllene oxide
19.359	557,134	22.69	1E + 06	12.61	3.18	203.1	0.084	2H-Cyclopropa[g]benzofuran
24.024	2E + 06	100	9E + 06	100	25.2	68.1	0.161	Neophytadiene
24.835	637,992	25.98	2E + 06	25.56	6.44	81.1	0.157	3,7,11,15-Tetramethyl-2-hexadecen-1-ol
25.411	1E + 06	42.45	4E + 06	42.52	10.71	81.1	0.161	3,7,11,15-Tetramethyl-2-hexadecen-1
31.064	468,188	19.07	1E + 06	13.53	3.41	67.1	0.1	(E,E)-Methyl linolelaidate
31.215	637,675	25.97	2E + 06	23.16	5.84	79	0.147	11,14,17-Eicosatrienoic acid, methyl ester
31.506	858,095	34.94	3E + 06	32.32	8.14	71	0.167	Hexa-hydro-farnesol
**(B)**								
6.591	131,777	4.41	392,385	2.78	0.95	44.1	0.09	Monomethyl malonate
9.35	609,951	20.43	1,481,562	10.5	3.57	95.1	0.107	endo-Borneol
9.966	501,142	16.79	2,336,018	16.55	5.64	110	0.248	Pyrocatechol
10.395	3E + 06	100	14,111,231	100	34.05	120	0.224	Coumaran
12.592	1E + 06	46.29	7,022,887	49.77	16.94	150	0.204	p-Vinylguaiacol
15.294	2E + 06	54.56	5,540,018	39.26	13.37	95.1	0.131	Alpha santolina alcohol
18.613	430,440	14.42	794,117	5.63	1.92	91	0.067	Humulen
19.373	2E + 06	73.74	5,020,261	35.58	12.11	203.1	0.084	Cyclopropa[g]benzofuran
39.64	2E + 06	56.68	4749427	33.66	11.46	149	0.107	1,2-Benzenedicarboxylic acid
**(C)**								
15.702	5,894,271	48.37	13,241,763	37.21	5.13	93.1	0.094	Humulene
18.331	6,998,724	57.44	17,735,706	49.84	6.87	79.1	0.1	beta-Caryophyllene epoxide
18.857	12,184,878	100	35,584,370	100	13.79	67.1	0.131	Humulene oxide
19.269	5,604,173	45.99	19,882,595	55.87	7.7	67.1	0.131	Caryophyllene oxide
22.179	4,861,002	39.89	18,735,011	52.65	7.26	109.1	0.147	Drimenol
25.448	3,237,750	26.57	12697359	35.68	4.92	81.1	0.147	3,7,11,15-Tetramethyl-2-hexadecen-1-ol
26.778	5,389,458	44.23	19,824,080	55.71	7.68	74	0.144	Methyl palmitate
31.118	4,228,256	34.7	14,158,473	39.79	5.49	67.1	0.124	Methyl linoleate
31.276	6,219,883	51.05	22,845,516	64.2	8.85	79	0.214	9,12,15-Octadecatrienoic acid, methyl ester
**(D)**								
6.553	475,471	6.94	1,106,068	5.59	2.31	99	0.141	M-Pyrol
18.328	405,020	5.91	800,942	4.05	1.67	79.1	0.07	beta-Caryophyllene epoxide
18.837	582,101	8.49	1,480,419	7.48	3.09	67.1	0.124	Humulene oxide
20.458	417,424	6.09	1,254,667	6.34	2.62	55.1	0.127	Globulol
25.438	640,981	9.35	2,449,409	12.38	5.12	81	0.164	3,7,11,15-Tetramethyl-2-hexadecen-1-ol
26.704	2E + 06	22.92	5,009,852	25.32	10.47	74	0.127	Methyl palmitate
30.639	1E + 06	18.56	3,462,672	17.5	7.23	67.1	0.107	9,12-Octadecadienoic acid, methyl ester
30.776	2E + 06	24.25	5,004,849	25.3	10.46	79	0.121	Methyl linolenate
31.034	571,769	8.34	1,820,926	9.2	3.8	71.1	0.144	Phytol
**(E)**								
9.356	6E + 06	64.28	14,136,851	26.65	14.21	95.1	0.131	Borneol
10.408	9E + 06	100	53,040,523	100	53.32	120	0.352	Coumaran
12.601	5E + 06	53.22	17,351,644	32.71	17.44	150	0.238	p-Vinylguaiacol
14.296	337,994	3.85	770,318	1.45	0.77	111	0.077	Cyclohexanebutanal
15.287	1E + 06	15.93	4,429,002	8.35	4.45	95.1	0.124	alpha-santolina alcohol
15.786	1E + 06	14.16	2,865,277	5.4	2.88	91	0.124	1(7),5,8-o-Menthatriene
18.127	305,613	3.48	561,559	1.06	0.56	91	0.067	8-Isopropenyl-1,3,3,7-tetramethyl-
18.599	439,654	5.01	976,253	1.84	0.98	79.1	0.087	Bicyclo[3.3.0]octan-2-one, 7-ethylidene
19.235	689,601	7.86	1,354,090	2.55	1.36	67.1	0.077	Caryophyllene oxide
19.366	1E + 06	13.11	3,986,649	7.52	4.01	203.1	0.124	Aristolone

***A)** Crude methanolic extract (Ph.Cr), **B)** Ethyl acetate fraction (Ph.EtAc), **C)** n-Hexane fraction (Ph.Hex), **D)** Chloroform fraction (Ph.Chf), **E)** Butanol fraction (Ph.Bt)*.

### *In-vitro* urease inhibition potentials

Ureases are implicated in the development of numerous pathological conditions of human being. They are major contributors in pathogenesis caused by *H. pylori* by allowing this bacteria to survive at acidic pH of the stomach thus leading to gastric, peptic ulcer and even cancer (Mobley and Hausinger, 1989). They are also involved in the formation of infectious stones and play a part in the pathogenesis of pyelonephritis, urolithiasis, hepatic encephalopathy, hepatic coma, and urinary catheter encrustation (Mobley et al., 1995). Similarly, in agriculture elevated urease activity may lead to considerable environmental and economic problems through the release of abnormally large quantity of ammonia into the atmosphere during urea fertilization. This persuade plant damage principally by depriving them from their vital nutrients, cause ammonia toxicity and raise pH of the soil (Mobley et al., 1991; Zonia et al., 1995). Consequently, the inhibition of urease producing organisms is among the important strategies for the treatment of infections caused by these bacteria, to diminish environmental pollution and augment efficiency of urea nitrogen uptake by plants (Andrews et al., 1986). So, the study of urease inhibition has medical, environmental and agronomic significance.

In our current study, Ph.Lo and Ph.Sp exhibited highest urease inhibition property causing 79.33 and 75.83% enzyme inhibitions at 1 mg/ml concentration with IC_50_ of 90 and 98 μg/ml, respectively. Standard drug thiourea showed 87.40% inhibition at the same concentration and IC_50_ of 80 μg/ml. Activity of Ph.Lo and Ph.Sp were comparable with positive control at the same concentration, which warrant further purification and characterization for possible utilization in gastric disorders. Ph.Chf and Ph.EtAc have also showed 62.16 and 52.16% enzyme inhibitions and IC_50_ of 520 and 880 μg/ml, respectively (Table 2). These fractions may be subjected to column chromatography for isolation of novel bioactive compounds.

**Table 2:** Urease inhibitory potentials of *Polygonum hydropiper* extracts and saponins, and essential oil.

**Samples**	**Concentration (μg/ml)**	**Percent inhibition**	**IC_50_(μg/ml)**
Ph.Cr	1,000	35.66 ± 0.44^***^	1,380
	500	24.50 ± 0.28^***^	
	250	18.00 ± 0.57^***^	
	125	11.66 ± 0.66^***^	
	62.5	9.53 ± 2.25^***^	
	31.25	5.00 ± 0.57^***^	
Ph.Hex	1,000	40.00 ± 0.57^***^	1,400
	500	33.83 ± 0.44^***^	
	250	27.50 ± 0.50^***^	
	125	21.33 ± 0.57^***^	
	62.5	18.66 ± 0.00^***^	
	31.25	13.50 ± 0.00^***^	
Ph.Chf	1,000	62.16 ± 0.44^***^	520
	500	51.16 ± 0.60^***^	
	250	43.50 ± 0.28^***^	
	125	37.00 ± 0.57^***^	
	62.5	31.00 ± 1.15^***^	
	31.25	26.66 ± 0.66^***^	
Ph.EtAc	1,000	52.16 ± 0.44^***^	880
	500	40.83 ± 0.44^***^	
	250	35.00 ± 0.50^***^	
	125	28.00 ± 0.00^***^	
	62.5	21.00 ± 1.90^***^	
	31.25	15.86 ± 1.10^***^	
Ph.Bt	1,000	25.33 ± 0.33^***^	2,390
	500	21.00 ± 0.00^***^	
	250	17.00 ± 0.28^***^	
	125	11.00 ± 0.00^***^	
	62.5	8.50 ± 1.00^***^	
	31.25	5.33 ± 0.33^***^	
Ph.Aq	1,000	38.16 ± 0.72^***^	>3,000
	500	25.00 ± 0.50^***^	
	250	11.83 ± 0.44^***^	
	125	8.00 ± 1.00^***^	
	62.5	5.00 ± 1.15^***^	
	31.25	2.00 ± 0.00^***^	
Ph.Sp	1,000	75.83 ± 0.44^***^	98
	500	70.00 ± 0.57^***^	
	250	67.00 ± 0.50^*^	
	125	56.66 ± 0.0^***^	
	62.5	44.33 ± 3.8^***^	
	31.25	38.66 ± 2.2^***^	
Ph.Lo	1,000	79.33 ± 0.66^ns^	90
	500	70.50 ± 0.57^***^	
	250	62.00 ± 0.00^***^	
	125	55.83 ± 0.44^***^	
	62.5	48.00 ± 1.00^***^	
	31.25	39.00 ± 1.15^***^	
Thiourea	1,000	96.29 ± 0.50	80
	500	87.40 ± 1.50	
	250	70.45 ± 0.00	
	125	66.66 ± 1.9	
	62.5	55.53 ± 0.0	
	31.25	44.33 ± 3.8	

*Result expressed as % inhibition (mean ± SEM of n = 3) and IC_50_ values. Values significantly different as compare to positive control*,

* *P < 0.05*,

*** *P < 0.001*.

### Anti-proteus activity

**Figure 2:**
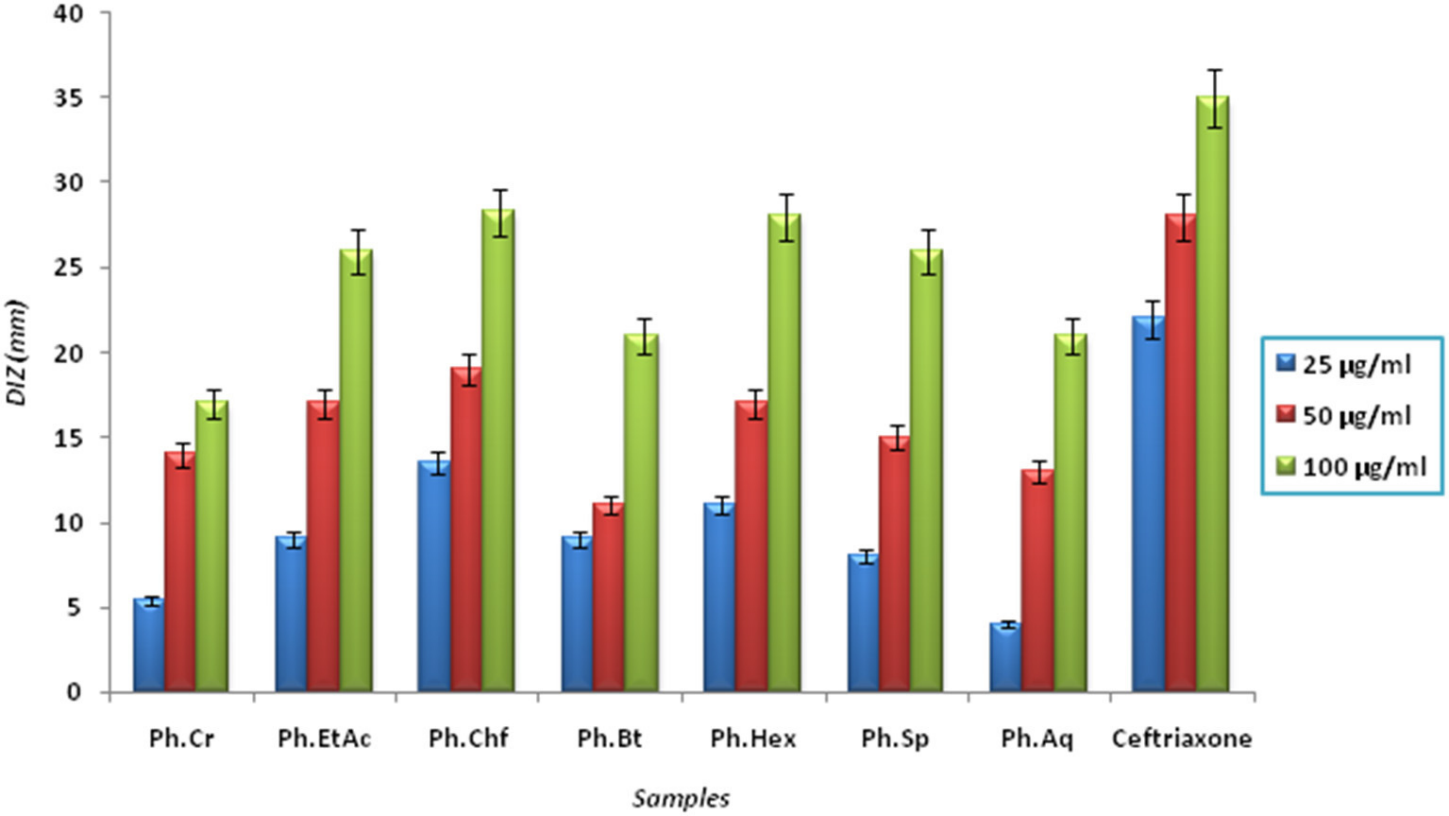
Diameter of inhibitory zone (DIZ) of different samples against *Proteus mirabilis* in disc diffusion assay.

**Figure 3:**
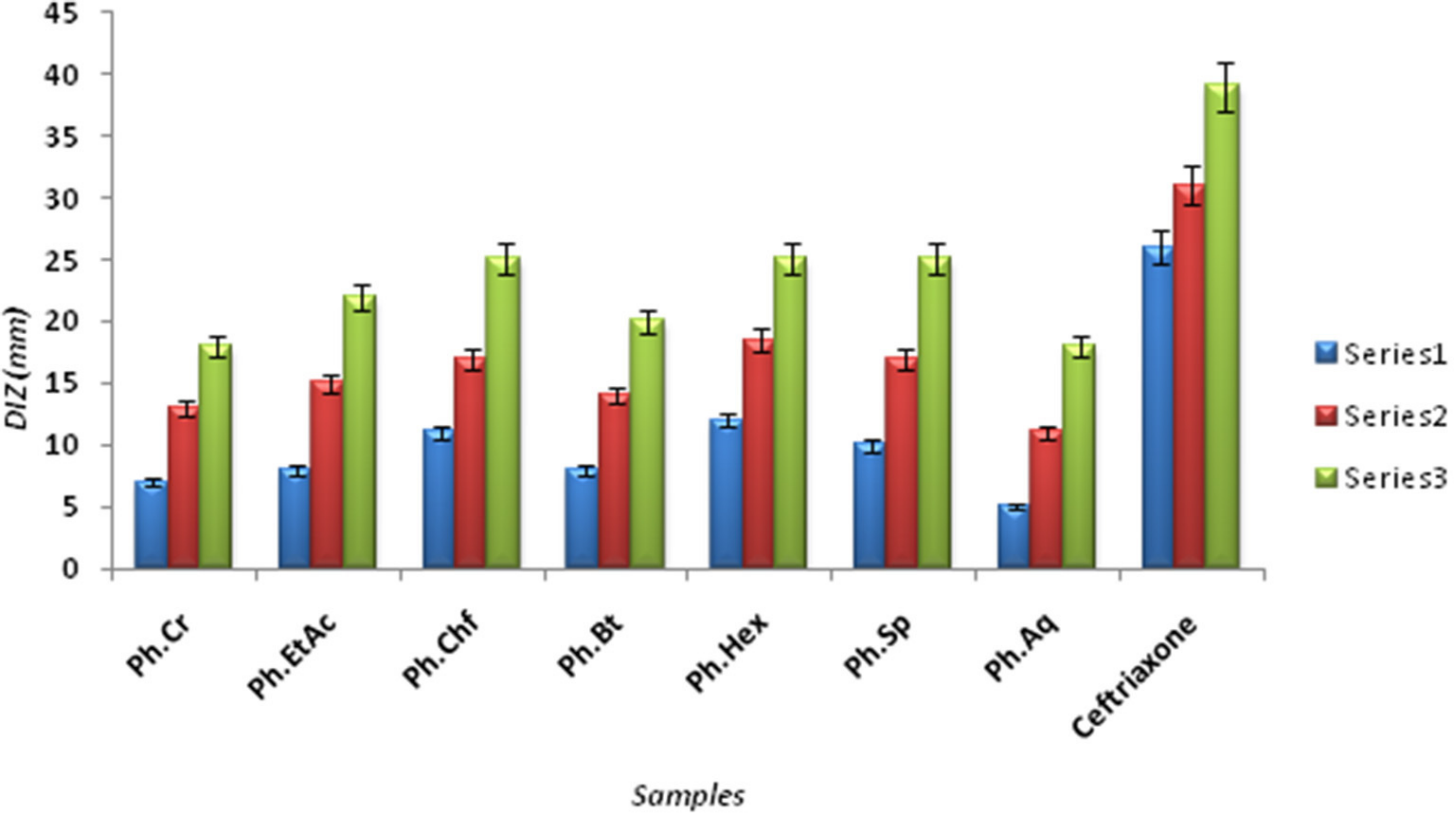
Diameter of Inhibitory zones (DIZ) in mm of different samples against *Proteus mirabilis* in Well diffusion assay.

**Figure 4:**
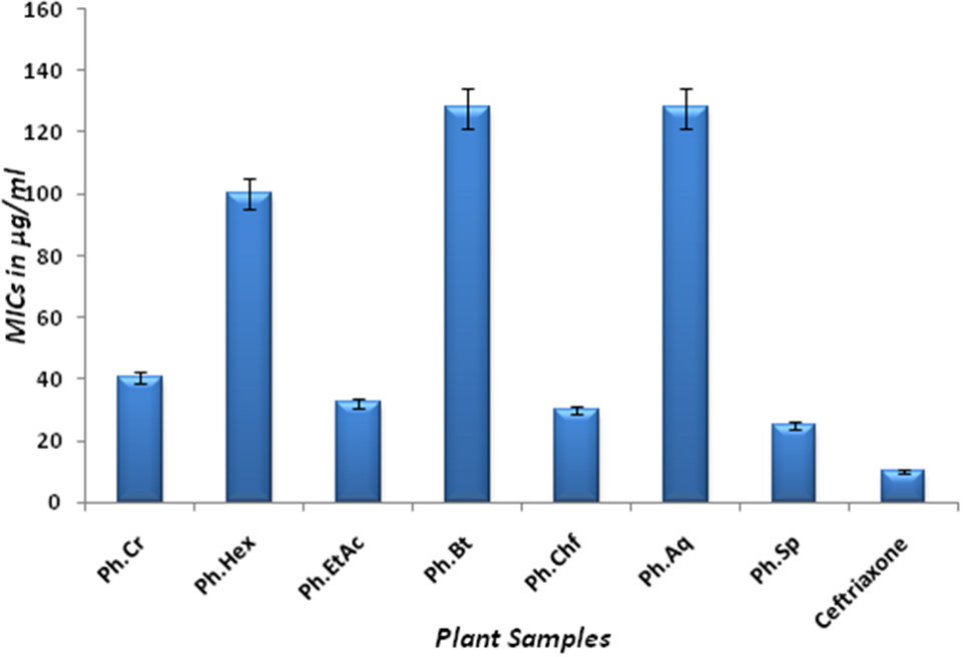
MICs of different fractions against *Proteus Mirabilis*.

The Bacterium *P. mirabilis* is a frequent cause of urinary tract infections, especially in the elderly catheterized individuals, and is the second leading cause of urinary tract infections after *Escherichia coli* (Mohammed et al., 2014). Ureolytic activity of numerous microorganisms including *P. mirabilis, Proteus vulgaris*, and *Ureaplsma urealyticum* is implicated in urolithiasis which cause chronic pelvic and kidney inflammation (Williamson, 2001). The ammonia produced by urease is responsible for the raise in urine pH followed by formation of struvite and apatite stones which are characteristic features of *P. mirabilis* infection. Excessive ammonia fabrication by these microorganisms may contribute to ammonia encephalopathy or hepatic coma and facilitate *H. pylori* survival in the stomach followed by infections (Sujoy and Aparna, 2013). Additionally, elevated level of ammonia disrupt mucosal permeability, especially hydrogen ions transport via mucosal surface and thus cause peptic ulcers (Suzuki et al., 1992). Consequently, research is focused on urease inhibitor compounds for the development of drugs useful against urease mediated bacterial infections (Mobley and Hausinger, 1989; Mobley et al., 1995; Estiu and Merz, 2004). In the current study, Ph.Chf, Ph.Hex, Ph.EtAc, and Ph.Sp fractions were found most active against *P. mirabilis* causing inhibitory zones of 28.25, 28, 26, and 26 mm, respectively at 100 μg/ml concentrations in disc diffusion assay (Figure 2). In well diffusion assay, each of Ph.Chf, Ph.Hex, and Ph.Sp produced inhibitory zone of 25 mm at 100 μg/ml concentration (Figure 3)”) Further, Ph.Sp, Ph.Chf, Ph.EtAc, and Ph.Cr were found most active exhibiting MICs of 25, 30, 32.25, and 40.50 μg/ml, respectively (Figure 4).

**Figure 5:**
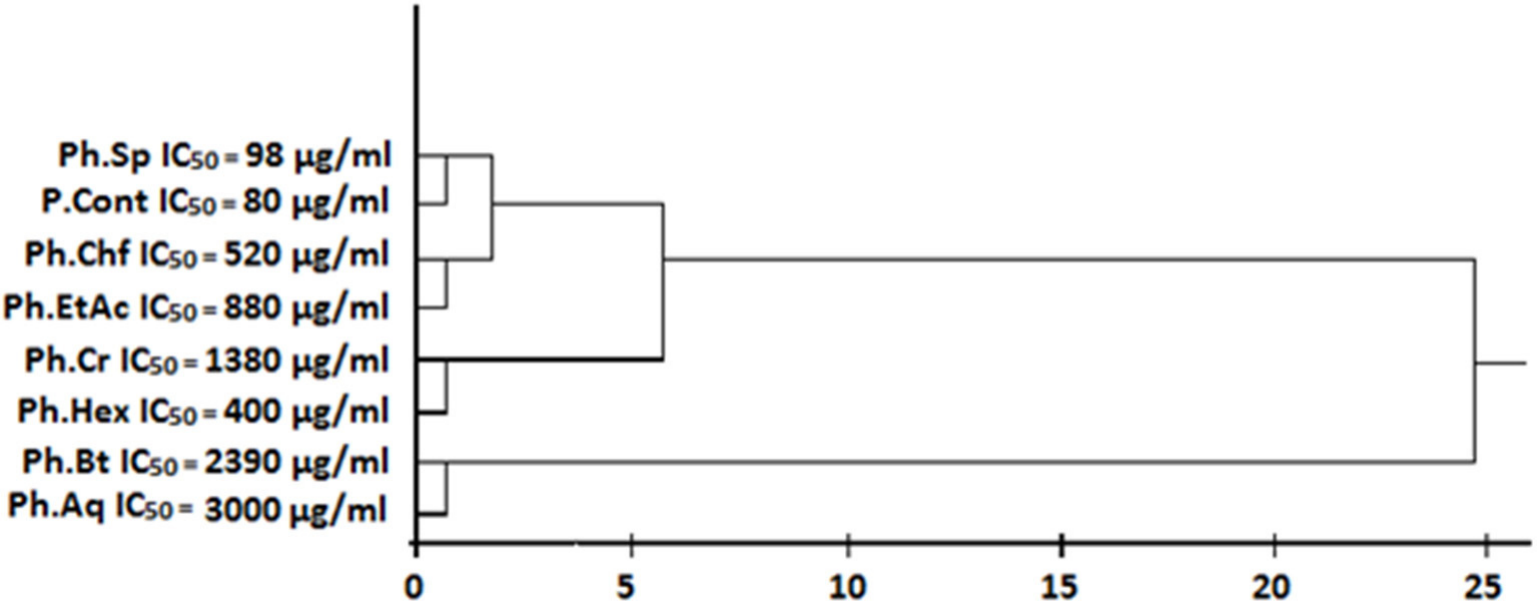
Cluster analysis and dendrogram based on IC_50_ of different samples in urease inhibitory assay.

### Gastric juice analysis

The aspirin induced ulcerogenesis in pylorus ligated rat model was associated with significant changes in the mean ulcer score [*F*_(5, 30)_ = 7.141, *P* = 0.0002], gastric juice volume [*F*_(5, 30)_ = 8.245, *P* < 0.0001], gastric juice pH [*F*_(5, 30)_ = 5.715, *P* = 0.0008], free acidity [*F*_(5, 30)_ = 4.544, *P* = 0.0033], total acidity [*F*_(5, 30)_ = 2.740, *P* = 0.0373], and pepsin concentration [*F*_(5, 30)_ = 2.335, *P* = 0.0664]. As shown in Table 3, treatment with aspirin at a dose of 200 mg/kg (Group II) for 4 days produced ulcerative changes in the gastric mucosa which were observed as drastic changes in the ulcerative biochemical profile in which aspirin significantly increased the ulcer score (*P* < 0.001), increased the volume of gastric juice (*P* < 0.001), decreased the pH of gastric juice (*P* < 0.001), increased the free acidity (*P* < 0.01), total acidity (*P* < 0.05), and pepsin concentration (*P* < 0.05) in the gastric juice as compared to vehicle treated controls (Group I). The prophylactic treatment with the Ph.Cr extract exhibited marked gastroprotective effect in the aspirin induced pyloric ligation ulcerogenesis model as the tested doses of 100 mg/kg (Group IV), 200 mg/kg (Group V), and 400 mg/kg (Group VI) afforded significant anti-ulcerogenic proclivity evidenced by a non-significant changes in the ulcerative biochemical parameters except at a dose of 100 mg/kg (Group IV) in which a transient increase in the ulcer score (*P* < 0.05) and a decrease in the gastric juice pH (*P* < 0.05) was observed compared to the vehicle treated controls (Group I). The positive control, ranitidine at a dose of 50 mg/kg (Group III) exhibited a robust gastroprotective profile as it is devoid of any aspirin like gastric ulceration observed as no obvious changes in the ulcerogenicity parameters.

**Table 3:** Gastroprotective effect of Ph.Cr extract in the aspirin induced ulcerogenesis in pylorus ligated rat model.

**Groups**	**Ulcer score**	**Gastric juice volume (mL)**	**Gastric juice pH**	**Free acidity (mEq/L)**	**Total acidity (mEq/L)**	**Pepsin (μg/mL)**
Vehicle	0.167 ± 0.1054	2.345 ± 0.2451	3.030 ± 0.2026	25.50 ± 2.262	42.17 ± 3.301	9.625 ± 0.5186
Aspirin (200 mg/kg)	3.500 ± 0.6055^***^	5.007 ± 0.5237^***^	1.360 ± 0.1699^***^	42.67 ± 3.896^**^	59.67 ± 5.439^*^	13.19 ± 1.068^*^
Ranitidine (50 mg/kg)	1.167 ± 0.3575	2.782 ± 0.2709	2.563 ± 0.3546	28.50 ± 3.149	46.67 ± 3.584	10.75 ± 0.9471
Ph.Cr (100 mg/kg)	1.917 ± 0.5069^*^	3.513 ± 0.3279	1.880 ± 0.1688^*^	33.00 ± 2.852	51.67 ± 3.051	11.06 ± 0.9297
Ph.Cr (200 mg/kg)	1.333 ± 0.4773	3.110 ± 0.2832	2.337 ± 0.2714	29.50 ± 2.643	48.50 ± 3.423	10.14 ± 0.8003
Ph.Cr (400 mg/kg)	0.917 ± 0.3005	2.642 ± 0.2670	2.663 ± 0.2813	27.33 ± 2.333	43.33 ± 3.913	10.02 ± 0.6426

*Values expressed as mean ± SEM*.

* *P < 0.05*,

** *P < 0.01*,

*** *P < 0.001, compared to vehicle treated control (one-way ANOVA followed by Dunnett's post-hoc test), n = 6 rats per group*.

### Histopathological examination

**Figure 6:**
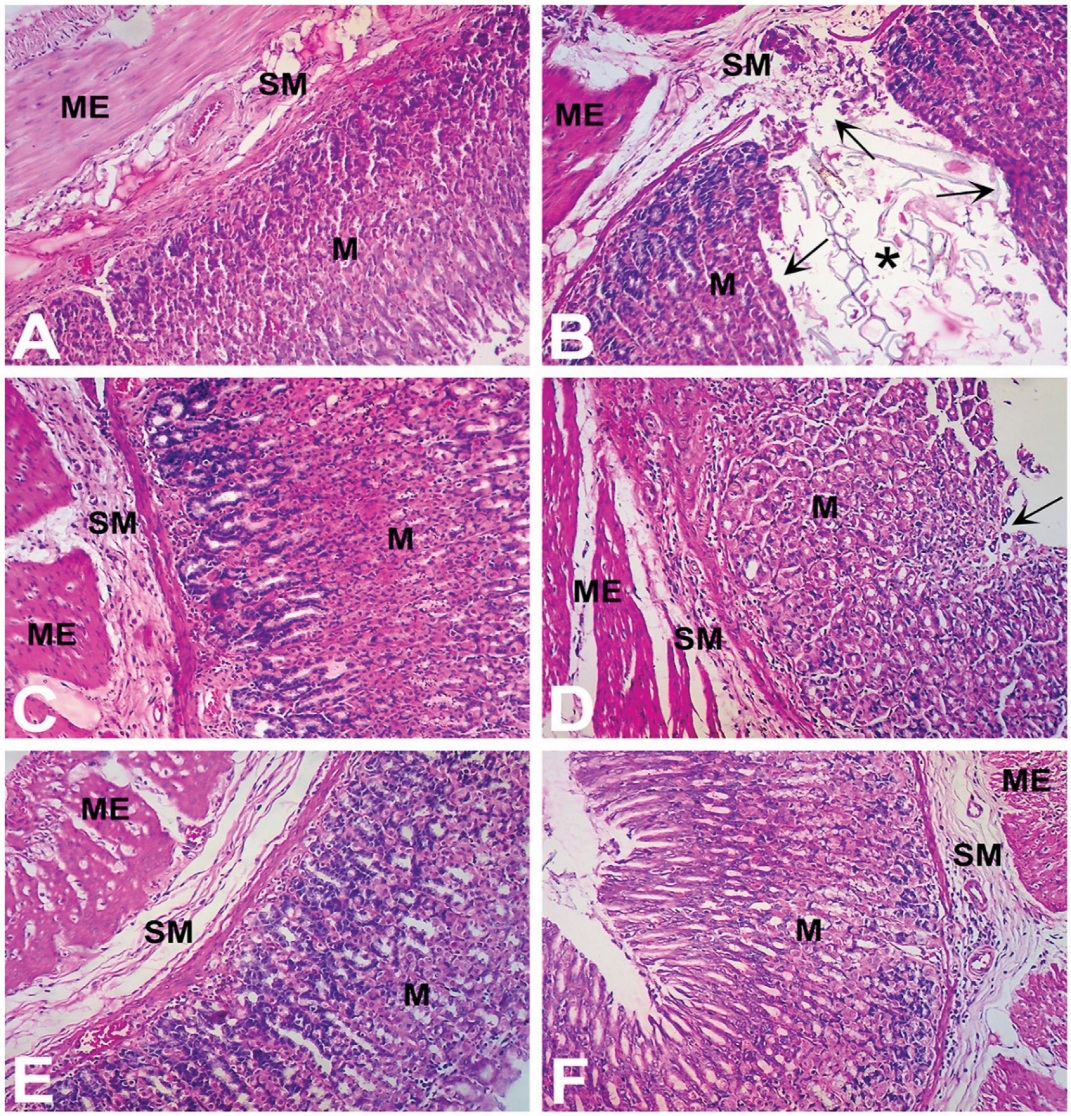
Gastroprotective effect of Ph.Cr extract in the aspirin induced ulcerogenesis in pylorus ligated rat model (H & E staining, ×100 original magnification) (*n* = 6 each). **(A)** Photomicrograph of a section of glandular portion of stomach from a vehicle treated control rat showing normal histological appearance of mucosa (M), submucosa (SM), and muscularis externa (ME). **(B)** Photomicrograph of a section of stomach glandular tissue from a rat treated with aspirin (200 mg/kg) showing disruption and erosion (arrows) of mucosa (M) which extend to the submucosa (SM) and muscularis externa (ME) along with necrotic debris visible in the lumen (asterisk) and base of the ulcer. Normal histoarchitecture of gastric mucosa (M), sub-mucosa (SM) and muscularis externa (ME) was observed in groups of rats treated with **(C)** positive control, ranitidine (50 mg/kg), **(D)** Ph.Cr extract at 100 mg/kg except for mild superficial shredding of surface mucosal epithelium (arrow), **(E)** Ph.Cr extract at 200 mg/kg, and **(F)** Ph.Cr extract at 400 mg/kg.

Treatment with aspirin (200 mg/kg) was associated with severe histopathological changes evidenced by numerous microscopic ulcers throughout the gastric mucosa. The pathological changes significantly altered the normal histoarchitecture of the glandular portion of the stomach and were observed as disruption and erosion of the mucosal epithelium. There was shredding of mucosal epithelial cells with necrotic debris observed in the lumen of the ulcers. The underlying blood vessels supplying the superficial mucosa were heavily congested with red blood cells. Severe infiltration of lymphocytes was observed in the connective tissues above the muscularis mucosae and throughout the damaged mucosa. There were marked edematous changes in the submucosal layer. In some areas, the mucosal layer was superficially eroded while in other areas the ulcer extended to the submucosa and down to the muscularis externa producing severe inflammatory changes in these tissue layers. A representative photomicrograph of gastric mucosa from a rat treated with aspirin (Group II) is shown in Figure 6B.

The histological examination of the gastric mucosa following treatment with vehicle (Group I; Figure 6A), ranitidine (Group II; Figure 6C) and the Ph.Cr extract at the tested doses of 200 mg/kg (Group V; Figure 6E) and 400 mg/kg (Group VI; Figure 6F) revealed normal histoarchitecture with no surface epithelial disruption, deep mucosal damage, edema, necrotic lesion, or leukocyte infiltration except for the 100 mg/kg dose of the Ph.Cr extract (Group IV) at which mild shredding of the mucosal surface epithelium was observed (Figure 6D).

## Conclusions

In the current study, *P. hydropiper* extracts, saponins and leaf oil exhibited significant urease inhibitory and anti-Proteus activities. Significant anti-ulcer and gastroprotective potentials were observed for Ph.Cr in animal model. Several compounds were identified in GC-MS analysism which can be responsible for gastroprotective potentials of the plant extracts and essential oil. Based on these results saponins were subjected to purification and characterization while Ph.Cr, Ph.Chf and Ph.EtAc were selected for activity-guided isolation of potent molecules. Our findings regarding gastroprotective and anti-diabetic potentials of *P. hydropiper* may provide scientific justification for further studies and folkloric uses of the plant.

## Author contributions

MA designed the project, processed plant collection, fractionation, literature review, experimental work, data collection, and manuscript drafting. MJ and FU acted as project supervisors, helped in project design, data analysis, and polished the research article. AS performed GC-MS analysis, compounds identification and manuscript revision. IU, WA, and NS helped in statistical analysis, data presentation and drafting the final version of manuscript. MS performed statistical analysis and helped in drafting the manuscript. All authors read and approved the final manuscript for publication.

### Conflict of interest statement

The authors declare that the research was conducted in the absence of any commercial or financial relationships that could be construed as a potential conflict of interest.
