# Corrigendum: GC-MS analysis and gastroprotective evaluations of crude extracts, isolated saponins, and essential oil from *polygonum hydropiper* L.

**DOI:** 10.3389/fchem.2023.1229054

**Published:** 2023-06-21

**Authors:** Muhammad Ayaz, Muhammad Junaid, Farhat Ullah, Abdul Sadiq, Muhammad Shahid, Waqar Ahmad, Ihsan Ullah, Ashfaq Ahmad, Nawazish-i-Husain Syed

**Affiliations:** ^1^ Department of Pharmacy, University of Malakand, Chakdara dir, Pakistan; ^2^ Department of Pharmacy, University of Peshawar, Peshawar, Pakistan; ^3^ Department of Pharmacy, Sarhad University of Information Technology, Peshawar, Pakistan; ^4^ Department of Pharmacy, University of Swabi, Swabi, Pakistan; ^5^ Department of Pharmacology, University College of Pharmacy, University of Punjab, Lahore, Pakistan

**Keywords:** *Polygonum hydropiper*, *Canavalia ensiformis*, indophenol method, urease, ulcerogenesis, *Proteus mirabilis*

In the published article, there was an error in Figure 6. The correct figure and its caption appear below.

**FIGURE 6:**
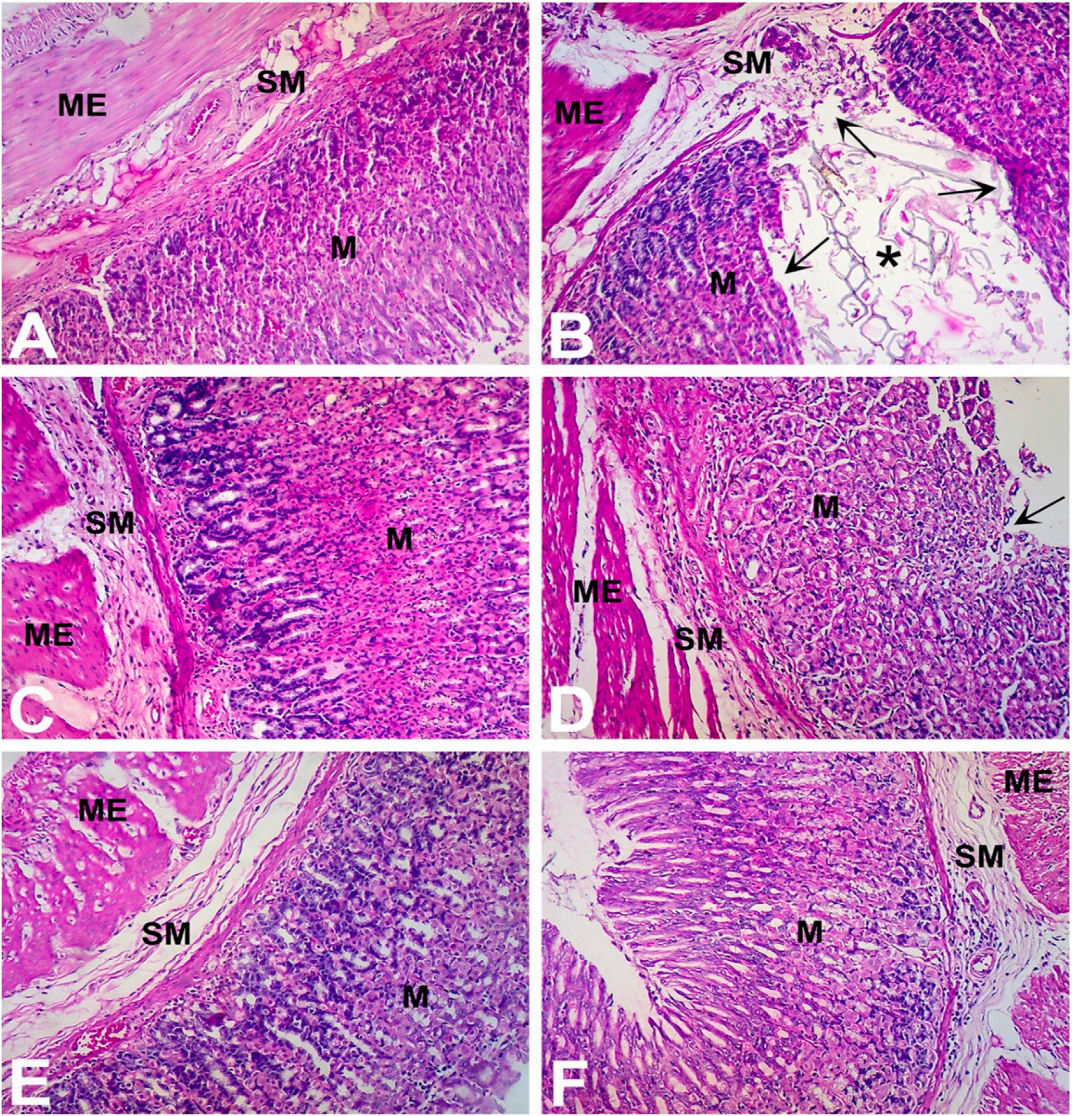
Gastroprotective effect of Ph.Cr extract in the aspirin induced ulcerogenesis in pylorus ligated rat model (H & E staining, ×100 original magnification) (*n* = 6 each). **(A)** Photomicrograph of a section of glandular portion of stomach from a vehicle treated control rat showing normal histological appearance of mucosa (M), submucosa (SM), and muscularis externa (ME). **(B)** Photomicrograph of a section of stomach glandular tissue from a rat treated with aspirin (200 mg/kg) showing disruption and erosion (arrows) of mucosa (M) which extend to the submucosa (SM) and muscularis externa (ME) along with necrotic debris visible in the lumen (asterisk) and base of the ulcer. Normal histoarchitecture of gastric mucosa (M), sub-mucosa (SM) and muscularis externa (ME) was observed in groups of rats treated with **(C)** positive control, ranitidine (50 mg/kg), **(D)** Ph.Cr extract at 100 mg/kg except for mild superficial shredding of surface mucosal epithelium (arrow), **(E)** Ph.Cr extract at 200 mg/kg, and **(F)** Ph.Cr extract at 400 mg/kg.

The authors apologize for this error and state that this does not change the scientific conclusions of the article in any way. The original article has been updated.

